# Narrative Devices: Neurotechnologies, Information, and Self-Constitution

**DOI:** 10.1007/s12152-020-09449-1

**Published:** 2020-09-28

**Authors:** Emily Postan

**Affiliations:** The University of Edinburgh School of Law, Edinburgh, UK

**Keywords:** Identity, Narrative, Neurotechnology, Neurodata, Cognitive privacy, BCI, Neuroimaging, Information governance

## Abstract

This article provides a conceptual and normative framework through which we may understand the potentially ethically significant roles that information generated by neurotechnologies about our brains and minds may play in our construction of our identities. Neuroethics debates currently focus disproportionately on the ways that third parties may (ab)use these kinds of information. These debates occlude interests we may have in whether and how we ourselves encounter information about our own brains and minds. This gap is not yet adequately addressed by most allusions in the literature to potential identity impacts. These lack the requisite conceptual or normative foundations to explain why we should be concerned about such effects or how they might be addressed. This article seeks to fill this gap by presenting a normative account of identity as constituted by embodied self-narratives. It proposes that information generated by neurotechnologies can play significant content-supplying and interpretive roles in our construction of our self-narratives. It argues, to the extent that these roles support and detract from the coherence and inhabitability of these narratives, access to information about our brains and minds engages non-trivial identity-related interests. These claims are illustrated using examples drawn from empirical literature reporting reactions to information generated by implantable predictive BCIs and psychiatric neuroimaging. The article concludes by highlighting ways in which information generated by neurotechnologies might be governed so as to protect information subjects’ interests in developing and inhabiting their own identities.

## Introduction

Neurotechnologies are used ever more widely in healthcare, research and by consumers, as well as in commercial, military, education and judicial settings. These uses involve the monitoring, collection, analysis and generation of increasing quantities of information about the states and functioning of our brains and minds. This discussion advances the claim that our encounters with these kinds of information can affect our identities in ways that warrant serious ethical attention. This claim is underpinned by my central argument that information about our own brains and minds can provide important tools in our construction of coherent, inhabitable identities – self-conceptions that equip us to make sense of and navigate our embodied lives. In doing so, this discussion fills a gap in debates about the ethical concerns and governance priorities raised by neurotechnologies and the information they generate. These debates currently focus chiefly on the ways that those other than the subject of the information may access and (ab)use this information. I suggest that this emphasis on others’ uses neglects, and may contribute to misunderstandings about, the ethical significance of whether and how we ourselves are able to access it and our interests in doing so. This gap must be filled if the governance of neurotechnologies is to respond appropriately to the full range of pertinent interests. This discussion addresses one aspect of that gap by offering a conceptually and normatively robust account of the roles of information about our brains and minds in our construction of our identities, and the nature of our identity-related interests in our encounters with this information. The focus here on the identity effects of information distinguishes this inquiry from those that explore the direct impacts of the physical presence or operation of neurotechnologies themselves on users’ self-conceptions.^1^ The present inquiry contributes to the less well-trodden ethical landscape of users’ interests arising from their encounters with the information generated.

In the discussion that follows, I first introduce the scope of the kinds of information and technologies with which I am concerned, before describing the gap in neuroethics debates that this discussion addresses. In order to fill this gap, and to explain why it is important to do so, I outline an account in which our identities are constituted by our own narratives about who we are. In doing so I will highlight both the embodied nature of our identity-constituting narratives and the conditions that apply if these narratives are to support us in leading our embodied lives. From these conceptual and normative foundations, I then build my central proposal: that information about our brains and minds can play important roles in our construction of coherent and inhabitable identities in ways that give rise to ethically significant interests. I illustrate this account with examples drawn from empirical literature reporting patient’s reactions to information generated by implantable predictive neurodevices and psychiatric neuroimaging. In concluding, I highlight some of the ways that information generated by neurotechnologies might be governed in ways that respond to information subjects’ interests in developing and inhabiting their own identities.

## Categories of Technology and Information

This discussion is intended to apply to information about individual users’ brains and minds, collected and generated by neurotechnologies used in a wide range of applications and settings.^2^ I will talk about ‘information about the brain and the mind’ as a pair, while taking a non-reductionist view of the mind which holds that, even if mental events have neural correlates, they are not wholly reducible to these. And knowledge of someone’s brain activity is not equivalent to knowledge of the qualities or contents of their thoughts or experiences [6]. While distinguishing information about the brain and mind, it is nevertheless useful to consider them as a pair here insofar as ethical concerns about handling of brain data are often grounded in concerns about the inferences that may be drawn from these to the health and functioning of our minds [7].

When using the term ‘neurotechnology’ I will be referring to those technologies that either collect data from users’ brains, or collect other kinds of data, for example physiological or behavioural, from which inferences about users’ brains or minds (or some combination of the two) are then drawn. These technologies include structural and functional neuroimaging and brain-computer interfaces (BCIs). BCIs function by acquiring signals from the brain using implanted or external electrodes and relaying these to an external device where these are analysed to produce outputs, such as movement of a cursor on a screen [8]. The arguments and recommendations to be made here are also intended to apply to technologies such as wearable and mobile devices that use physiological and behavioural data such as heart-rate or online activity to make inferences *to* the states or functions of the brain or mind, for example, indicators of mental health status. The purposes for which neuroimaging and BCI technologies may be used vary widely. These include monitoring, prediction, diagnosis, targeting and delivering therapeutic interventions, device control, communication and entertainment, in a range of research, clinical, judicial, commercial, military, educational, and consumer settings [9–11]. Various methods of structural and functional neuroimaging – ranging from magnetic resonance imaging (MRI) to cheaper, more portable, less spatially-precise methods such as electroencephalography (EEG) – are used in clinical contexts, for example to identify neural damage associated with trauma or dementia [10]. Neuroimaging is also widely used in research, both to address health-related aims and to contribute to basic understanding of neural functions, cognition, senses, affective responses and behaviour [11]. Applications of neuroimaging technologies also extend beyond clinical and research contexts. For example, it is used in researching consumer preferences [12] and in the justice system (particularly in the United States), for example to provide mitigating evidence of structural anomalies or (more rarely and controversially) as a form of ‘lie detection’ [13]. Some commercial services offer direct-to-consumer (DTC) neuroimaging, purporting to diagnose mental health conditions [14].

Uses of BCIs span a similarly broad spectrum. Investigative applications of BCIs using implanted electrodes include assisting paralysed individuals to communicate or move objects [8]. Implanted BCIs are used in healthcare to detect neural events associated with disease symptoms (one experimental example of which is discussed further below) and in delivering therapeutic interventions [15]. Non-invasive BCIs are also widely available as consumer products. These include, for example, EEG headsets that are used to control devices for gaming or personal computing, or to provide data for health and wellbeing wearables that purport, in conjunction with associated mobile apps, to allow users to monitor brain activity, or seek to modulate it [16].

The pertinent shared feature of the technologies to be discussed here is their capacity to generate information about the user’s brain or mind in forms that are (or could in principle) be accessible by that individual. When referring to the ‘information generated’ here I intend this to include data collected by these technologies, as well as the products of analysis of these data by the technologies themselves, or by researchers, healthcare professionals or technicians overseeing their use. As such, this discussion applies to information about neural activity and structural features, as well as to inferences drawn from these about the user’s mental and physical health, autonomic functions, motor functions, and his (or her) wider wellbeing. These might include information about past events (such as a stroke, or a pattern of poor quality sleep), about current states of affairs (such as a diagnosis of advancing Alzheimer’s disease, or indicators of stress and anxiety), or predictive information (such as warnings of an impending seizure, or an elevated risk of developing a major depressive disorder).

These kinds of information, and how they are communicated, will vary considerably in the extent of analysis and interpretation involved, and in the richness of their semantic content – ranging from a warning light on a device signalling seizure risk, to a detailed clinical diagnosis. They will also differ in their epistemic qualities – that is, in the extent to which they engender meaningful, reliable and accurate beliefs in the individual about their brain, mind, or associated capacities and characteristics. While the central argument of this article is intended to apply to the kinds of information that can plausibly be produced by neurotechnologies now (or in the near future), rather than their more speculative and hyped capacities (such as their abilities to read the content of our thoughts [17]) there is still considerable scope for variability in the specificity and reliability of the kinds of information that fall within its purview. This is attributable not only to the different stages of development of many of these technologies and the techniques and standards used for collecting, analysing, validating and communicating data, but also because in many cases the information produced will be probabilistic [18, 19]. The relevance of variation in information quality will become apparent below.

## From Others’ Uses to our Own Encounters

There are at least three pressing high-level reasons why it is necessary to address how information subjects’ interests may be affected by information generated by neurotechnologies about their brains and minds. First is the sheer quantity of information generated by neurotechnologies – attributable both to the proliferation of devices and applications as well as the high levels of data extraction integral to their functions [7]. Secondly, there is considerable variation in how these kinds of information are governed, who may have access to them and for what purposes. These matters are subject to different regulatory regimes depending on whether, for example, the information counts as personal data for the purposes of data protection law, is part of patient records, classed as research findings, or is commercially sensitive – each carrying different implications for information subjects’ expectations and entitlements to information generated.^3^ In some instances (such as health-tracking wearables) direct communication of information to the user is integral to the purpose and function of the device, but this is not the case for all devices [4]. And, for example, it is not inevitable that researchers will provide participants in neuroscientific studies with resultant findings relating to their own circumstances, beyond perhaps serious and actionable health risks [20]. Such variation is not surprising, given the scope of uses and settings involved, but it does give rise to questions about whether information subjects’ interests are appropriately protected across all these domains. Addressing this question requires identification of the full range of significant interests likely to be engaged, as well as attention to where the rules, functions and practices governing the (non)disclosure of information to users fail to recognise and protect these interests. Thirdly, there is a tenacious, but far from self-evident, assumption that information about our brains and minds is particularly closely related to, and uniquely revealing of who we are [21, 22]. This unexamined assumption cannot to do the explanatory and normative work often demanded of it in neuroethics debates.

Given these three factors, the generation and management of information generated by neurotechnologies are not a novel areas for ethical concern. But these factors also explain why it is necessary to ensure this territory is well-mapped and that any relevant and ethically significant interests are properly identified and characterised. It is here that I wish to suggest that there is an important gap to be filled. Ethical discussions concerning information gathered and generated by neurotechnologies tend to be dominated by questions about how parties – other than information subjects themselves – might (ab)use it for research, healthcare, public health, commercial, criminal justice, administrative, or surveillance purposes (for example see [23–25]). Consequently, concerns relating to information subjects’ interests tend to focus on the validity and clinical utility of the information, appropriate justifications and consent for the kinds of third party uses just listed, and anxieties about invasions of their privacy and consequent discrimination or stigmatisation, rather than the impacts that information subjects' *own* encounters with and uses of this information might have directly on them, let alone on their identities.

The specific gap this discussion seeks to fill is the absence of serious and well-characterised ethical attention to how information subjects’ encounters with information generated by neurotechnologies may impact on their capacities to develop, make sense of and inhabit their identities. By ‘identity’ here I mean that which characterises us as individuals and provides the foundations for our practical engagement with the world and other people. For example, the impacts of information access on our interests in self-constitution are not addressed in Marcello Ienca’s and Roberto Andorno’s call for a suite of new ‘neuro’ rights to address the threats posed by emerging neurotechnologies [26], in the ‘*Neuroethics Guiding Principles for the NIH BRAIN Initiative*’ [27], or the OECD’s 2019 ‘*Recommendation of the Council on Responsible Innovation in Neurotechnology*’ [28]. And they are only briefly alluded to amongst 2016 UN Global Neuroethics Summit’s guiding questions for international brain initiatives [29]. In notable contrast, the potential impacts of genetic information on our identities has attracted considerable, though not always unproblematic, ethical and regulatory attention [30–33].^4^

Ethical concerns about how others may use information about us are legitimate ones. Those relating to privacy and unauthorised access may be particularly pressing. This is due not only to the potentially clinically or personally significant, sensitive, or stigmatising nature of information about our neurological and mental health or cognitive capacities and functions, but also to the fact that neurodata may (like genomic data) be sufficiently rich and detailed to be inherently identifying of individuals and disorders [23]. This kind of ‘numerical identification’ of the source of information with a known individual is important, but not the sense of identity with which I am concerned in this discussion.^5^ And, although the ways that others judge, categorise and treat us based on what they infer from information about us may well affect our identities in the ‘self-conception’ sense in which I *am* interested, these indirect identity impacts are not the focus of the present discussion inasmuch as the aim here is to shine a light on the ethical significance of individuals’ own encounters with information about themselves. Over the following sections I will propose and illustrate a normative conception of the role that our own encounters with information about our brains and our minds may play in our development of our identities.

I do not wish to imply that the possible impacts of users’ access to information generated by neurotechnologies on their self-conceptions have been wholly neglected in neuroethical debates. Potential identity effects have been discussed in relation to neuro-related wearables and in empirical studies of clinical applications of fMRI and implantable BCIs (see, for example, [9, 35–38]). Some of these studies are discussed further below. However, I will suggest that these existing accounts would benefit from more robust conceptual and normative foundations if suggestions about consequences for identity are to have purchase and be taken seriously in information governance decision-making. Specifically, these analyses do not always offer clear accounts of what is meant by identity, what roles information about the brain and mind might play in our identities, or why these roles are sufficiently significant to attract ethical attention. My aim here is to provide these missing foundations. In the following sections I will say more about the particular conception of identity on which I will focus, examine how encounters with information about our brains and minds may contribute to or undermine identities thus conceived, and explain why these impacts *matter*, in order to ground my central claim – that the identity impacts of our encounters with information about our brains and minds warrant serious ethical attention.

## A Normative, Embodied, Narrative Conception of Identity

I want to suggest that applying the lens of identity as constituted by a particular kind of embodied first-person narrative provides a means for appreciating the ethical significance of the role of information about our brains and minds in our development and occupation of our identities. I will propose a strongly normative account of narrative identity-constitution, which seeks to explain why narrative changes *matter* and engage important moral identity-specific interests.^6^ Narrative conceptions of identity have achieved particular prominence in debates about the identity effects of deep brain stimulation (DBS) [39–41]. Here, however, I am applying it to the impacts of information rather than physical interventions.

As noted above, in talking about identity here I am not (directly) addressing questions of numerical identity – that is, questions of sameness and persistence of entities. My focus is upon identity in, what Marya Schechtman terms, the “characterization” sense [42 p.1]. That is, the sense we are concerned with when we inquire what someone is *like*, whether their behaviour is *characteristic* of them, or whether an experience has changed them. And this concerns the totality of who someone is, not merely unitary social identifiers or labels – though these may be part of the picture. The concept of identity I am referring to is also a *practical* one [43]. That is, I shall take it that someone’s identity is much more than an inert descriptor. It provides their ‘first person perspective’ and the foundations for their interpretations and evaluations, motives, life projects and relationships, and thus the framework and conditions for their agency and engagement with the world [44].

Following writers including Schechtman and Catriona Mackenzie I will take it that our identities in this practical, characterisation sense are constituted by our self-told stories of who we are [42, 44]. These stories are woven from our experiences and characteristics, what we have done and plan to do, our attitudes and values, our roles, and our relationships to others. They are *narratives* in that they are temporally extended and, critically, because they are not simply receptacles of everything that we experience. Rather they are selective, the product of interpretation and editing. And, as such, they are continually evolving in response to new experiences and interpretive contexts. I will also follow Schechtman and Mackenzie in holding that our self-constituting narratives are first-personal.^7^ This is because the sense-making and unifying nature of narrativity, and the role of our self-narratives in grounding our evaluative and practical capacities (as described further below) are premised on a single (albeit complex and evolving), internally-referential perspective of the particular individual who interprets, judges and acts. Which is not to say that our identities are, or can be, constructed in isolation, or that we can never be confused or mistaken about our characteristics. On the contrary, self-constitution, on this conception, is an inherently relational, “dialogical” activity [46, p33]. And our relationships, roles and others’ views of what we are like contribute to and constrain our first-personal accounts of who we are [47].

Narrative self-constitution theories are not without detractors [see, for example, 48, 49]. However, I would suggest that features of narrativity, such as plotting, coherence or fragmentation, capture the phenomenology of self-conception, what we care about when we desire to be a particular kind of person, and what we fear about losing our sense of who we are. And, as Mary Walker argues, narratives offer the kind of “epistemological strength” that we attribute to our identities – the capacity to make sense of ourselves and our experiences and explain why we acted as we did, or why we value particular things [50, p.66]. I suggest that these are exactly the kinds of experiences and capacities we might have in mind if we are concerned or optimistic about how information about our brains our minds could affect how who we understand ourselves to be.

One possible objection to narrative theories is that, if our identities are *constituted* by our self-told stories, this might imply that we are free to invent whatever kind of identity we wish, no matter how fanciful [49]. However narrativity here is a normative not just a descriptive concept. We do not want to have *just any* identity narrative, but to develop a narrative *worth having*. It is this normativity that introduces limits on what can function as identity-constituting narrative and thus precludes unfettered self-invention, or at least its desirability [42, 44, 51]. The suggestion here is that an identity narrative that serves us well in in leading a fulfilling existence will, firstly, be one that supports our interpretive, evaluative, capacities and practical engagement with the world. Agency and autonomy are amongst these capacities, but by no means exhaust them. They also include our abilities to make sense of who we are and our place in the world, to make judgements about what is valuable or admirable, to plan, and to sustain long-term projects, commitments and relationships. And, secondly such a narrative will be one that provides us with a recognisable and inhabitable sense of who we are. By this I mean one that allows us to ‘live with ourselves’, that includes meaningful roles, and that is itself intelligible to us while also supporting us in making sense of and navigating our lives. These qualities of coherence, intelligibility and inhabitability alone may not be sufficient for leading a rich and fulfilling life. Nevertheless, I would suggest that the capacities, attributes and experiences they support are they relatively uncontroversial aspects of desirable human existence, in that we tend to value them, and they reflect the kinds of elements commonly cited by as important by ethical theories that seek to enumerate universal and objective conditions for wellbeing [52, 53]. However, not just any form or substance of narrative will be capable of supporting these qualities, and it is here that we encounter both the constraints on what comprises a worthwhile practical identity and the basis for our identity-related interests.

Narratives capable of supporting these desirable features will, I suggest, exhibit four qualities. The first is that the narrative is internally intelligible and coherent, making sense on its own terms, so that the individual has a reasonably clear sense of who they are and a firm, reasonably integrated (both diachronically and synchronically) foundation from which to interpret, judge, act.^8^ The second is that it is reasonably intelligible to others with whom the individual lives and interacts. The third is that the narrative reflects the individual’s experiences of and encounters with their own embodied states, abilities and (mal)functions, their environment and relationships to others. The fourth is that that the kinds of self-descriptors of which the narrative comprises will, as far as possible, be ones the individual welcomes or, at least, does not find wholly alienating or unbearable.^9^ Each of these constraints may be met to a greater or lesser extent, reflecting the fact that we have only limited control over our circumstances and that life inevitably introduces disruptive, hard-to-comprehend, and unwelcome plotlines and we each have differing capacities to accommodate these in coherent and inhabitable stories.

The first three of these requirements owe much to Schechtman’s proposed twin constraints upon identity-constituting narratives: that they must be amenable to “articulation” and “cohere with reality” [42 p.114, p.119]. However, Schechtman justifies the second of these constraints with reference only to the need for our identities to be recognisable to others to allow us to function in social relationships. She does not acknowledge the significance of our materiality to our self-narratives, that we are also beings who think, sense, feel and act in embodied ways.^10^ Because of these features, I suggest, there is a parallel need for our self-narratives to be responsive to the “basic contours of reality” so that they are also intelligible and inhabitable *to us* in the context of our material, embodied lives [42 p.123].^11^ The fact that our self-narratives, are inescapably embodied has been recognised and developed by several theorists including Mackenzie, Kim Atkins, and Mary Walker [40, 55, 56]. Here I seek to go beyond the conceptual foundations these authors offer, to make claims about the ethical significance of the epistemic and interpretive roles that information about our brains and minds can play in constructing and inhabiting embodied identities.

In asserting that our identity-constituting narratives are embodied I mean to make several interdependent descriptive and normative claims. First, at the most basic level, our stories of who we are will inevitably contain plotlines about our bodies and embodied minds and be shaped and mediated by our experiences of these – for example, our health, senses, gender, or reproductive status. Secondly, our *capacities* to construct intelligible and inhabitable self-narratives may be enabled or constrained by aspects of our embodiment, for example, our cognitive capacities or mental health, or how comfortable or alienating we find particular bodily characteristics [57].^12^ Thirdly, a critical aspect of developing and maintaining a coherent and inhabitable practical identity involves constructing a self-narrative that remains sustainable and intelligible when confronted by our embodied experiences, capacities and limitations – including those of our brains, cognition, senses, moods and health – while also providing a framework that supports us in making sense of and navigating our experiences and encounters with these. And while our narratives will inevitably be disrupted by and evolve in response to new embodied experiences, we benefit to the extent that they can also weather these experiences (or at least quotidian ones) without deep or permanent harm to their intelligibility, inhabitability or coherence.

The key normative implications of what I have said in this section are that our identities are not just something we have by default. Rather, as their authors, we are active in developing them. And they serve us well insofar as they exhibit the coherence, intelligibility and inhabitability that underpin our sense of who we are, our practical and evaluative capacities, and our unique perspectives the world. The degree to which they exhibit these features will vary and, to this extent, our projects of self-constitution may fare better or worse. However, even though we are the authors of our own narratives, the success of our identity projects (whether they fare better or worse) depends not only on our own abilities as storytellers. It also depends on the ways that our lived contexts, which include our brains and embodied minds, support or hinder our efforts. And – because narration is inherently an interpretive endeavour – the construction of a coherent, inhabitable self-narrative will also crucially depend on the epistemic and hermeneutic tools we have available to us to help us make sense of, give meaning to and prioritise these lived experiences.

## Neurotechnologies as Sources of Narrative Tools

My central claim in this paper is that information about the states and functions of our brains and minds generated by neurotechnologies can provide precisely the kinds of epistemic and hermeneutic tools just mentioned – tools that contribute to the content of our identities, but also, crucially, to the extent that they ‘fare better or worse’. This information fulfils this latter role not because we are essentially our brains (or even our minds), nor because neuro-information provides privileged insights into who we ‘really are’, but because our brains and embodied minds are part of the inescapable context of our lives, and thus of our stories of who we are. Fresh, or otherwise inaccessible, information about these contexts can assist us in the interpretive tasks of developing and maintaining these stories. At perhaps the most straightforward level, then, my claim is that information generated by neurotechnologies may supply new or revised modes of self-description to our identity narratives.^13^ For example, incidental findings from a routine MRI scan may reveal that someone is a stroke survivor; a research study investigating correlations between neural activity and susceptibility to psychiatric illness may generate findings that indicate that a participant is at significantly increased risk of major depressive disorder; or a wearable fitness monitor may display data telling the user that they are a particularly light sleeper. The individuals concerned may or may not then incorporate these new self-descriptors into their self-narratives, potentially modifying or replacing existing ones.

Moving beyond this, the information generated by neurotechnologies could contribute by indirect means, by instigating a course of action or changing our perspectives in ways that then feed into our narratives. For example, confirmation of a diagnosis of Alzheimer’s disease by a structural brain scan might lead a patient to re-evaluate their priorities, bring forward a long-deferred project, or participate in clinical research into dementia. Or a user of a recreational BCI neurofeedback device might seek to re-evaluate their work patterns and attitudes towards parenting upon observing that they receive on-screen indications of relaxed and meditative brain state more readily at the end of a day spent with their family than at their workplace.

Similar kinds of information could also serve the coherence and inhabitability of these narratives by filling in gaps, reframing, or offering context and explanations for the kinds of experiences we have as beings that lead embodied, thinking, feeling lives and thus providing us with ways of anticipating, understanding or navigating these. For example, we might imagine a situation in which receiving a diagnosis of an autism spectrum disorder based in part on functional neuroimaging data could help someone to understand and feel more confident in the atypical ways they view the world, and to feel solidarity with others with similar diagnoses. Or data gathered by a wearable device indicating poor sleep might allow someone to make sense of recent changes in their moods and inability to cope with their workload.

A narrative conception of identity offers a way of understanding how each of these examples of potential information-initiated changes and reinterpretations can be seen as much more than instances of acquiring new social identifiers or altering our behaviours. As part of a wider self-narrative they represent potentially far-reaching – beneficial or detrimental, welcome or unwelcome – edits to or (re)interpretations of an individual’s identity. My central claim here is that these changes are ethically significant insofar as they contribute to the inhabitability, intelligibility and coherence of our self-narratives. This is because we, and the qualities and richness of our lives, are benefitted or harmed in non-trivial ways by the extent to which we are able to make sense of who we are and navigate our lives accordingly. For these reasons, we can have significant interests in whether and in what ways encounter information about our brains and minds.

Before looking at two examples to highlight the nature of these interests in more detail, I wish to address four possible misconceptions about the nature of the claim made above.

First, in arguing that our self-narratives are essentially embodied I am not asserting that we are, or should be, *defined* by the characteristics and functions of our brains or minds, nor that we are obliged to incorporate all and any available information about them wholesale into our self-narratives.^14^ As with any aspect of our lives, such inclusions will be selective and interpretive. Exclusion of, even prominent aspects of our neural functions, health or mental states from our narratives, can be part of intelligible self-constitution.

Secondly, my claim is not that information derived from neurotechnologies is unique in fulfilling the kinds of narrative roles described above. In other contexts, different kinds of information about our bodies, biology or health – for example, genetic, reproductive or physiological – could occupy parallel roles.^15^ Indeed, my proposal that much of the ‘identity value’ of information from neurotechnologies lies in its instrumental, sense-making or contextualising potential, leaves space to recognise how other types of bioinformation could perform similar functions in relevant circumstances.^16^ Empirical studies provide some indication as to why our encounters with and experiences of our brains and minds, and thus neuro-information, might neverthless perhaps be particularly likely to be viewed as significant to our identities. Some of this may be attributable to cultural conceptions of the brain as the seat of the self, and (problematic) popular representations of the powerful insights of neurotechnologies, for example to reveal our ‘true’ traits [61, 62]. Some of it may also be to do with the “special significance” of brain, and injury or illness affecting it, due to the potentially grave and pervasive implications its functioning for our lives [63, p6]. Some studies, however, indicate participants hold equivocal views about such significance [22]. It is also possible that the perceived precision, quantifiability, and objectivity of information generated by neurotechnologies plays a role [36]. This is a fruitful area for further investigation.

However, my intention here is not to make exceptionalist claims about the identity-significance of information from neurotechnologies, but rather to highlight that the potential impacts of these technologies on users’ identities, and associated identity-related interests, extend to the information they generate in ways that warrant appropriate characterisation and serious ethical attention. Moreover, the aim is to clarify that these interests obtain while eschewing *both* exceptionalist *and* essentialist assumptions about the nature or contents of our identities, by explaining why such interests need not – indeed, should not – be premised on attributing the identity-significance of neuro-information to either the achievement of a ‘complete’ identity, or its putative capacity to reveal superior or corrective insights into what we are ‘really like’ – which brings me to the third point of clarification.

The proposal that information generated by neurotechnologies could provide valuable context and explanations is not the same as claiming that it does so because it reveals the ‘truth’ about our identities’. For example, Walker has (sceptically) examined the possibility that some neuroscientific findings –for example, those that purport to indicate how our effective motives differ from our acknowledged ones – could be interpreted as presenting a challenge to the truth of our self-narratives, or offering correctives to these, by revealing where our ‘real’ characteristics depart from the stories we tell about them [50].^17^ According to a narrative conception of identity, however, there is no true identity that precedes and trumps our own accounts of who we are [44]. Subject to the constraints noted above, it is these accounts that constitute our real identities and it is with reference to them that we appropriately and meaningfully explain and characterise who we are. Moreover, the nature and meaning of our motives, attitudes and behaviours derive from the embodied and relational narratives of which they are a part, not from information about neural activity or, at least, not from this taken in isolation. According to the account I have offered here, the potentially valuable ‘explanatory’ power of neuro-information lies not in *revealing* our identities, but in providing contextual insights into our brains or minds that can assist us in our interpretive endeavours in constructing and making sense of our identities in light of our lived experiences.^18^

The suggestion that information generated by neurotechnologies can provide insights into who we ‘really are’ is, however, integral to claims that some neurotechnologies pose risks to “mental privacy” due to their putative (current or imminent) capacities to read our minds [23, 26, 65–68]. For example, Ienca and Andorno call for a new human right to mental privacy to provide legal protection against the involuntary revelation of users’ “intentions, views and attitudes” by neuroimaging technologies [26 p.3].^19^ Such concerns are often couched in a Cartesian-flavoured assumptions that our own minds are (and should be) private and inscrutable domains, to which we ourselves usually have direct and relatively uncomplicated access. This is reflected, for example, in Ienca and Andorno’s description of the mind as “an unassailable fortress” demarcated by the “boundaries of the skull”, and Paul Wolpe’s references to the mind as “humanity’s one impenetrable refuge” and “the privileged domain of my individual functioning” [26] p.1, p.2; 71 p.85, p.86]. Concerns about mind-reading by neurotechnologies are problematic, insofar as they rely on a neuro-reductive view of the mind. They also exemplify the ethical preoccupation, noted above, with how others, rather than information subject’s themselves, might use our neuro-information. And to the extent that they imply that ethical concerns relating to neuro-information and identity arise chiefly in relation to the risks of others’ gaining illegitimate insights into our ‘inner selves’, while our own encounters with this information do not raise comparable concerns as we already have direct access to our own minds, they risk being mis-directive as to the epistemic powers and potential value of neuro-information to information subjects. We do not have perfect or uncomplicated insights into the states, functioning or health of our brains and minds. But nor does such information open a window into the naked truth about who we are. Information generated by neurotechnologies can provide complementary and interpretive materials that help us to make sense of our experiences, and to locate these within or exclude them from our accounts of who we are, in ways that engage significant interests.

Fourth and finally, my aim is not to suggest that information about our brains and minds will necessarily make unequivocally welcome or valuable contributions to the inhabitability our identities. On the contrary, some might be neither welcome nor valuable. For example, someone might experience diagnoses of serious mental illness as distressing and stigmatising in ways that make their identity profoundly uncomfortable and difficult to inhabit, or as undermining self-descriptors or projects that are central to their identity. In some cases this might represent a profound and disruption to their sense of who they are. It is important, however, to distinguish unwelcome from detrimental effects of information here. Marked shifts in our sense of who we are may be welcome or unwelcome, valuable or detrimental.^20^ As I discuss further below, our interests do not necessarily lie in *preserving* the contents and shape of our identities in unchanging form. Rather, they lie in being in a position to maintain or restore – as far as possible – the coherence, intelligibility and inhabitability of our self-narratives through change and in response to the vagaries of lives, our bodies and minds, so that we are equipped us to understand who we are and what we value, and to engage practically with the world. Sometimes this will entail a change, even a marked one, in our self-conceptions. And information about our brains or minds may play an important role in sense-making through such changes. For example, a psychiatric diagnosis that is initially experienced as distressing and unwelcome might, for some individuals, eventually serve a valuable role by enhancing the coherence and inhabitability of their self-narratives by, for example, offering explanations for periods of psychological distress or disorientation [57].^21^

The value, desirability, or even pertinence, of particular information to someone’s identity narrative is not inevitable, and will depend on their particular circumstances, cultural environment and existing self-narrative. It will be clear from what I have said so far, however, that my position stands in contrast to a concerns raised by some, specifically in relation to information communicated by consumer neurotechnologies, that neuro-information might be intrinsically detrimental to self-understanding where its objectifying and material focus supplants the putatively more direct, phenomenologically rich and authentic insights of our own senses and introspection [9, 75]. If wholesale usurping of direct experience was to occur, such fears might be warranted but, short of this, I am proposing that quantified information about our materials selves can have a valuable complementary role to play, offering welcome additional insights, filling important explanatory gaps, or providing appropriate interpretive context [76].

There is, however, one kind of neurotechnology-generated information that is unlikely ever to make valuable contributions to our self-narratives, even if we welcome it. This is information that fails to provide accurate, reliable, or meaningful insights about the states or functioning of our brains or minds, or at least fails to do so without making these limitations clear and comprehensible. The risk lies in our using information of dubious epistemic quality to re-interpret or construct a self-narrative incorporating erroneous beliefs about these aspects of our lives. The concerns here are, first, that such a narrative will provide a poor foundation for interpreting and navigating our embodied experiences and, second, that an identity narrative constructed using these tools is rendered vulnerable to dissonance with future experiences. As such, unreliable information represents a dual threat to the coherence and inhabitability or our identities, which I discuss further below.

Having set the grounds for and mapped the boundaries of my central claim – that information about our brains and minds generated by neurotechnologies can provide important epistemic and hermeneutic tools in the construction of coherent, inhabitable, embodied identity narratives – I will now turn to look at two examples that illustrate it in greater depth. These examples are drawn from empirical studies in which the authors have explored (possible) identity impacts of two different applications of neurotechnologies. However, the authors’ analyses do not offer a unified underlying conceptual account of the roles that neuro-information may play in our identity narratives or, more importantly, to explain why these roles engage significant interests and, thus, why information disclosure decisions and methods are of moral significance for irreducibly *identity*-grounded reasons. The account I have proposed adds a critical layer through which to interpret these valuable findings and recognise their normative implications.

## Two Illustrative Examples

### Predictive Brain Implants

This example concerns BCI neural implants that monitor and analyse brain activity associated with epileptic seizures in users with a history of treatment-refractory epilepsy and warn them about the onset of seizures [77]. The user is alerted by visual or auditory signals on worn or handheld devices, giving them the opportunity to take medication or otherwise prepare. The following discussion focuses on the potential identity impacts of this kind of predictive information.^22^

One small qualitative study conducted semi-structured interviews with six participants in a trial of this kind of predictive BCI, to explore users’ experiences, including any postoperative impacts on their “sense of self” and whether using the device had changed who they are or their “sense of control” [38 p.11, p12, 78, 79]. The authors conclude that only two of the six participants recounted experiences that amounted to a changing (sense of) self. As described below, of the two they identify as having had such experiences, the authors’ analysis corresponds with several aspects of the theory-based proposals about the identity impacts of information in the preceding sections. However, valuable as these empirical insights are, I wish to suggest that the existing analysis reflects a somewhat restricted view of the nature and significance of identity impacts and the role of information in this. Without departing from the available evidence, it may be possible to offer more theoretically and normatively nuanced analyses with which to inform the ethical implications of uses of these kinds of neurotechnologies.

I will first address findings relating to two participants who reported experiencing impacts on their (sense of) self. The first of these participants reports that, “*I found myself changing…it changed my confidence, [the device] changed my abilities – it changed how stressed I was…With the device I felt I could do anything – I can do everything I wanted to do I was more capable of making good decisions…it gave me a new lease on life*” [38 p.6]. This participant also describes how her epilepsy had previously long been a source of anguish and “*an opposition to me*” [38 p.5]. The authors of the study, do not use the term ‘identity’ in analysing these findings, but it is implicit in their description of using the BCI as having “affected her aspirational goals and augmented her activities as part of the process of self-definition and self-expression” [38 p.8]. In doing so they echo the suggestions I have made above – that information about neural activity may enable the independence and control that are central to our constitution of our own identities. These findings also highlight ways in which identity changes can amount to more than mere epiphenomena or pleasing experiences. They can be practically efficacious, in an individual’s agency and their projects of self-building. One aspect that the authors’ analysis does not bring out so clearly, however, is the implication – signalled by the participant reporting that “*with this device I found myself*” – that predictive information supplied the BCI allowed the user feel more comfortable in her own identity as someone with epilepsy, and to reinterpret the meaning of being epileptic and how this colours other aspects of her identity such her decision-making [38 p.5]. The value of this outcome may be appreciated when viewed in terms of the importance of the inhabitability of our accounts of who we are.

In contrast, a second participant, who describes how she had previously not characterised herself as epileptic and “*pretended that* [her epilepsy] *didn’t really exist*”, reports that the alerts delivered by the BCI “*made me feel like I was just sick all the time…like I was different to everyone else*” and as if she “*didn’t have control over what* [she] *was going to do*” [38 p.7]. These findings usefully highlight the ways that information about our brains could be detrimental to our identities, imposing unwanted or stigmatising self-descriptors or by conflicting with our existing, preferred accounts of who we are. In analysing this participant’s views the authors interpret the negative experiences of the second participant as being rooted in a “dramatic clash” and lack of corroboration between the information disclosed by the device and “the patient’s self-image and self-narrative” [38 p.9].^23^ This indeed captures a crucial part of this information’s impact. However, they do not go further to inquire why such a conflict might be problematic in ways that go beyond feelings of distress or “loss of control” [40 , p.^9^]. Focusing on (even serious) affective changes and experiences of control alone risks overlooking further reasons why we may, or may not, have interests in preserving our existing sense of who we are or in being confronted by experiences that threaten this. In this example, the participant’s response indicates that the disruption to her existing self-conception is indeed contrary to her interests because it threatens her ability to ‘live with’ who she is and to have a intelligible sense of self that provides a firm foundation from which she can act and to engage with the world. But, as discussed below, preservation of an existing narrative in itself is not inevitably valuable.

The findings from this study make a vital contribution by highlighting the potential identity impacts of information generated by a particular neurotechnology. The authors analysis of their findings differs from one premised in ideas related to narrativity, as it is conducted through a lens in which ethically significant identity changed is rendered in terms of “self-estrangement” [38, p.^7^]. Self-estrangement is characterised as involving an abrupt and involuntary change in an individual’s qualitative experience of self, such that they are unable to access or identify with who they were beforehand [38, 80]. Estrangement is described as “deteriorative” when associated with unwelcome changes, distress and loss of control, or “restorative” when associated with welcome changes, including the individual’s perceptions of enhanced capacities or reinstatement of their ‘real self’ [38, p.^8^]. However, restorative estrangement is not presented as unequivocally beneficial insofar as it is associated with “distorted perceptions” and alienation from one’s “previous self” [ibid].

While recognising that self-estrangement may well capture the experiences of several participants in this study, I would like to suggest that an analysis framed in terms of narrative self-constitution could offer some alternative, or differently nuanced, ways of thinking about the impacts of this predictive information that draw out a wider range of identity effects and the nature of their ethical significance.

For example, the authors suggest that the first participant discussed above acquires a “new postoperative identity” becoming ‘estranged ‘from her former one and that, even though she welcomes this, it is nevertheless a cause for ethical concern [38, p.^7^]. However, a narrative lens invites us to query whether an even abrupt change in our experience of self necessarily represents either alienation from a former self, or something detrimental (or of ambivalent value). A narrative conception regards change as inherent to our identities.^24^ Even dramatic changes in how we see ourselves, our characteristics, and our values, may be consistent with a retaining a persisting, intelligible and inhibitable identity, provided we are able to tell a unifying story that incorporates the change and able to reflect upon and interpret earlier chapters in light of later ones [81]. Of course, this is exactly what estrangement precludes. However, I suggest that it is at least plausible that the testimony of the first participant could reflect a kind of acute, yet nevertheless intelligible narrative transition – rather than estrangement – and be valuable to her. Dramatic identity change may be positively in someone’s interests to the extent that it better equips them to make sense of who they are through altering circumstances or to navigate their experiences of living with a serious illness such as epilepsy. Of course, not all changes are innocuous let alone valuable. Some narrative disruptions may be experienced as ‘loss of self’ – for example as might accompany the onset of debilitating illness – and represent a significant identity-related harm. However, a narrative interpretation suggests that this harm does not attach to the sheer fact of disruption. Rather it obtains when disruption renders us unable to recognise ourselves, make sense of who we are, or find meaning in our lives, and to lose the interpretive framework and foundations from which we navigate and engage with the world [57].

Construing the ethical significance of information’s effects on identity solely in terms of self-estrangement, may also lead us to miss other consequences that warrant attention. For example, a third participant in the study reported that the device gave them “*more confidence*”, made them feel “*more in control*”, reduced their reliance on their relatives, and allowed them to “*push on*” to do what they “*wanted to do*” [38, 78]. The authors do not class this as a change in sense of self (indeed the participant themselves asserts that they had not changed “*as a person*”) [38 p.5]. However, without seeking to second-guess this testimony, within it we might recognise precisely these feelings of increased self-efficacy as non-trivial developments in someone’s story of who they are, while a reduced reliance on relatives reconfigures both the relational plotlines of their narrative and, possibly, the respective contributions of its co-authors.

Finally, integral to the idea of estrangement is the idea that the identity change is involuntary [80]. And the authors’ analysis conducted in these terms implies that the participants are passive with respect to the identity effects of the predictive information generated by their devices. While it is true that users cannot control when warning signals are generated, it seems not unreasonable to suppose that – unlike, for example, direct neural effects of DBS^25^ – some users can exercise agency in deciding how to interpret and use the information (or not) in constructing their accounts of who they are. This much is indicated by several participants in the study, one of who reports that they “just ignore[d]” the alerts, while another appears to enjoy the quotidian ubiquity of their device [38, p.^5^]. This not true of all participants. For example, one participant describes how the “constant beeping” made her feel like she “had no control” [38, p.^7^].^26^ These findings, viewed through the lens of narrativity, offer a valuable reminder that in some cases information from neurotechnologies may be experienced as an uncontrollable ‘impact’ on our identities, but in others it can offer a tool, facilitating our agency in our identity development. The example discussed next will highlight some further ways in which analysis of identity impacts of neuro-information through the lens of narrativity that may bring out the wider nature and ethical significance of these impacts.

### Psychiatric Neuroimaging

Several empirical studies have explored patients’ (actual or anticipated) reactions to receiving predictive or diagnostic information based on neuroimaging findings that purport to provide information about the structural or functional neural correlates of serious psychiatric conditions such as depression or schizophrenia [35–37, 82–84]. These findings include attitudes that may be read as indicating roles for this kind of information in patients’ identity narratives. For example, some participants in these studies report that they do (or would) welcome information about neural correlates of their symptoms as “*proof*” of the objective reality of their illness [36, 82]. They may view it, for example, as validating their suffering, allaying others’ scepticism [82], or demonstrating that they are not “*just crazy*” [36 p.74]. Other respondents anticipate that it might allow patients to reframe their illness as just another physical disease, thus reducing stigma and self-blame, motivating uptake of therapies [83, 84], or facilitating a separation between self and disorder. Clinicians interviewed in one study, however, anticipated more negative reactions, fearing that patients would view the implicit authority and objectivity of neuroimaging data as indications that the disease is more intractable, or lead to pessimistic identification with their apparently disordered brains [35].

These findings lend plausibility and texture to my core contention – that information generated by neurotechnologies may play roles in our self-conceptions that warrant ethical attention. Several of these studies use the language of identity to analyse some of their participants’ responses to neuroimaging information [35, 36]. However, they draw upon a relatively narrow conception of identity impacts and associated normative palette. For example, identity changes tend to be framed in terms of the role information may play in bolstering specific modes of (biologised) self-description, such as ‘being depressed’ or someone with defective brain chemistry [36, 37]. Identity benefits are, accordingly, seen in terms of reducing stigma. Meanwhile, identity harms are interpreted in terms of potentially stigmatising or demotivating effects of adopting a neuroreductive view of the self, for example as someone with “error in them” [35, p.^6^]. These analyses provide valuable insights. However, the identity-significance of other reported impacts – for example, those of explaining or publicly validating illness experiences, reducing self-blame, or making therapeutic choices – tend not to be interpreted or labelled as such. From the perspective of a narrative conception, we might interpret these reactions as indications of some of the ways that results from psychiatric neuroimaging may shape individuals’ identities – for better or worse – in a wider range of ways, for example by providing new evaluative lenses though which to interpret their experiences and their relationship to their illness, or by shifting how other people’s attitudes impact on their self-conceptions. The account I have proposed makes space for recognising identity impacts beyond the adoption or loss of labels. It also offers a means of articulating why both new labels and fresh explanations may engage not only clinical interests, but significant identity-related ones – beyond stigma – to the extent that they enhance or undermine individuals’ capacities to make sense of their experiences of psychiatric illness as part of their accounts of who they are, and inhabit these accounts as the vessels in which they navigate their own and others’ responses to illness.

These findings also offer (albeit indirectly) a means of appreciating the ways in which the positive and negative roles that neuro-information may play in our identity narratives are contingent not only upon the circumstances of individual recipients, but also upon the reliability and accuracy of the predictions, diagnoses or explanations that the information can provide. There are enduring questions about the (current) capacities of functional neuroimaging to provide reliable diagnostic or predictive information in clinical psychiatric contexts [85–87]. These doubts stem not only from maturity of the technology, but also from more fundamental concerns about the irreducibility of the mind (and its health) to the brain and the extent to which existing diagnostic categories are not coextensive with neurological differences. If a psychiatric patient were to use misleading or meaningless imaging findings in the construction of their identity, *without being aware of* its epistemic limitations, the concern is that this may not only have clinical implications – for example misdirecting treatment choices – but that it may undermine the coherence and inhabitability of their identity. For example, misdiagnosis may ill-prepare someone to anticipate, make sense of or manage the particular experiences of living with a particular disorder, while overreliance on neurological explanations may engender unwarranted fatalism, or lead someone to undervalue environmental or social contributions to their mental health. Even if these outcomes do not inevitably or immediately render someone’s identity incoherent, my suggestion is that they may contribute to a distorted frame through which the individual interprets their past, navigates the present and plans their future narrative, and renders this narrative vulnerable to challenge by future illness experiences.

Empirical studies such as those discussed above are crucial in illustrating and enhancing our understanding of the potential identity impacts of neuro-information. The findings cited indicate how information from two particular applications of neurotechnologies can affect users’ identities and do so in diverse ways depending on their circumstances and experiences of living with epilepsy or psychiatric illness. The narrative account I have presented in the preceding sections offers valuable additional means of interpreting and drawing out the nature and significance of these effects *qua* identity impacts (as opposed to solely effects on health or emotional wellbeing) and why identity changes may (or may not) engage important interests. It also provides some indications as to how these interests might be appropriately protected and promoted in practice.

## Practical Implications for Information Disclosure

Building on the argument and illustrative examples above, I now turn to propose some high-level indications of the ways in which I believe the claims made above should make concrete differences to ethical decision-making and practices governing whether and how we – as users and information subjects – access information about our brains and minds generated by neurotechnologies.

The central recommendation to be drawn from what I have argued is that possible impacts of information generated by neurotechnologies on users’ interests in developing coherent, intelligible and inhabitable identities must be included as part of ethical decisions about making this information available to them. These impacts need to be weighed alongside those on other more routinely considered interests – such as, health, avoidance of distress, autonomy, privacy and freedom from discrimination – when considering ethical implications about whether and how to convey this information. According to the characterisation of identity interests offered here, the roles that information about our brains and minds may play in meeting these are equivalent neither to mere curiosity, nor a contemporary desire to track and quantify, nor yet to relatively bounded interests in the acquisition or loss of particular discrete self-descriptors. Rather they are rooted deep in our capacities for self-understanding and for navigating and engaging with our embodied states, the world and other people. A such, these interests are far from trivial, they go to the very heart of our existences as embodied persons. While, they may intersect with aspects of the other more commonly considered interests listed above, they are not coextensive with or reducible to these. For example, I have suggested the coherence and inhabitability of our self-narratives matters in part because it provides the foundations for our capacity for autonomy – where autonomy requires critical, reflective evaluation and reconciliation of our motives and choices in light of our wider values and goals, developed and exercised in relational and embodied contexts [88, 89]. However, not only is autonomy just one aspect of the valuable practical capacities supported by our self-narratives, but attending to information’s effects on the coherence and inhabitability of our identity-narratives may demand a different set of considerations than attending autonomy alone does, when it comes to deciding what information to disclose or how to do so (depending on the conception of autonomy interests in operation).^27^ Similarly, finding oneself unable to make sense of who one is may very well be distressing but, as just indicated, it has wider implications that that contemporaneous experiences of distress. Meanwhile the path to a more coherent identity that better serves us in navigating our lives may not itself be without emotional burdens.

### Whether to Disclose

Questions about identity-sensitive disclosure practices are then in part questions about *whether* the information generated by neurotechnologies should be offered or communicated to those to whom it pertains. Importantly, according to the characterisation of identity interests offered here, this means recognising the potentially substantial identity *benefits* of information from neurotechnologies, while not ignoring the possibility of threats to identity. This is a significant departure from presumptions that our identities are either impervious to fresh information, or else vulnerable to being broken by it.

In some cases, information communication will be integral to the function of the neurotechnology in question – as in the case of the implanted predictive BCIs of the first example above. However, in other contexts, disclosure may not be standard practice and will require justification on grounds of recipients’ interests in knowing. For example, much ethical guidance on policies for returning individually-relevant research findings to participants prioritise the return of clinically significant and actionable findings [20].^28^ One might imagine research findings indicating evidence of several ‘silent strokes’ or unusual neural activity, generated as part of neuroimaging studies or BCI development that do not meet these criteria, but nevertheless exhibit potential identity significance, such as offering possible explanations for experiences of cognitive decline or mood changes, which could make valuable contributions to the participants’ self-narrative. Identity-significance alone may not be the only relevant factor in reaching disclosure decisions in such circumstances, but the claims made above illustrate why it should be weighed as an important consideration amongst others.

One key consideration in any decision about disclosure will be the quality of the information generated. I have suggested that misleading or unreliable information about our brains or minds is likely to run counter to our interests in constructing coherent, resilient identities. Attending to these interests adds a further layer to existing *health-related* concerns about providing poorly-evidenced and unreliable information to patients, participants, or consumers as expressed, for example, in relation to the risks of accurate health information and advice generated by consumer neurotechnologies [16, 19]. It also provides a countervailing position to one particular kind of argument – based on a somewhat thin conception of autonomy – that, where health-related risks are not serious or inevitable, individuals should be free to access information of questionable epistemic value from consumer technologies if doing so is enjoyable, serves their curiosity, or simply their choice.^29^ It is by no means obvious that these kinds of ‘personal utility’ engage more critical interests than concern for our abilities to develop coherent, inhabitable and resilient identities. I would, therefore, suggest that potential for identity harms may provide additional persuasive grounds for seeking to control the marketing of neurotechnologies or services that provide unreliable or erroneous information (or at least doing so without explicit explanation of these limitations). It also adds weight to the importance of ensuring the suitability of the algorithms and training data used in cognitive and mental wellbeing wearables and mobile apps to ensure that they provide accurate data and advice to all users [93, 94].

### How to Disclose

Thus far I have focused on what recognition of identity interests implies for decisions about *whether* to share or disclose information. But no less important is a focus on *how* information is conveyed. By this I mean to capture the explanatory and interpretive context in which it is communicated and the support that is offered to the recipient in understanding what it means (and, just as importantly, does not mean) for her own neurological, mental and cognitive health and wellbeing. There are a number of reasons why the context and manner of disclosure is so important.

One is the role of information quality in its potential identity impacts, as just discussed. It is possible that some of the risks of building misapprehensions into one’s self-narrative may be averted if recipients are made aware of its epistemic limitations.^30^ A second reason is that, as is apparent in the two illustrative examples offered above, whether a recipient experiences neuro-information as valuable or detrimental to their identity is idiosyncratic. It may not be possible to predict how any particular recipient will react. The way in which it is offered and conveyed could provide a critical opportunity to anticipate and manage negative reactions. Underlying each of these reasons is a more fundamental one. Identity development is in itself an essentially interpretive and relational undertaking. We often rely on others when finding meaning and significance in the myriad experiences and details of our lives and when determining what to use, include or reject from our accounts of who we are. The need for this support where neuro-information is concerned may be even more pressing than with more quotidian experiences given its novelty, technical complexities and possible sensitivities.^31^

If interpretive support is indeed central to promoting and protecting our identity interests, this has particular implications for implants and wearables that deliver information about neurological and mental health and wellbeing directly, without the intercession of a person and in a semantically minimal way, for example, simply as an alert, or in a quantified or graphical form. We need to consider very carefully the consequences of providing potentially identity-significant neuro-information through device interfaces that offer little or no opportunity for integrated or concurrent contextual information and interpretive support. In some cases, such as the predictive BCI described above, the immediacy of an unadorned alert serves an important unmet clinical need. Even in these cases, however, there is an accompanying need to manage users’ expectations and understanding of the information they will receive and provide appropriate sign-posting to further supplementary information and support. This may be more readily achieved in care and research settings than with consumer products.

Gilbert and co-authors recommend that, before inclusion in experimental trials of predictive BCIs, potential participants should undergo psychometric assessment of links between their self-image and epilepsy and susceptibility to identity-related harm, thus their suitability for inclusion in trials or particular needs for preparatory support [38].^32^ In light of what a narrative conception suggests about the idiosyncratic ways that neuro-information may affect our identities (for better or worse), not least because our existing self-narratives are likely to shape how we respond to and use fresh information, this is an interesting and pertinent recommendation. People will vary to the extent to which they and their self-narratives are vulnerable or resilient the impacts of this information. However, care should be exercised when it comes to designating any particular groups as inherently more or less susceptible to identity harm. Vulnerabilities to such harms may have multiple sources and be dependent on contextual factors beyond the individual [96]. Moreover, while the possibility of serious identity harm means that such pre-operative assessments may indeed be a sensible precautionary measure, I would repeat here the need for caution about conceiving of identity change, even abrupt and marked change, as necessarily something about which we should be concerned or seek to avert. All of our identities are in a state of perpetual evolution and reconfiguration. I have sought to highlight in this discussion that encounters with information about our brains and our minds may serve to reshape anyone’s account of who they are, and in ways that are not necessarily detrimental. And any of us may need support in making sense of information presented to us by neurotechnologies, whether this entails incorporating it or rejecting it from our understandings of who we are. If the coherence and inhabitability of our self-narratives are indeed disrupted by the advent of new information, what matters is that we have access to any available interpretive tools to reconstruct a sense of who we are that serves us in continuing to make sense of and navigate our embodied, thinking, feeling and perceiving lives.

## Conclusion

If identity impacts are to feature as serious considerations in bioethical debates about how to manage information generated by neurotechnologies – as I have argued they must be – and if users’ interests in their own encounters with this information are to be appropriately protected, then it is not enough for these debates to allude in broad terms to possible identity effects’ (or even ‘narrative identity effects’) of these encounters. We need to understand what these effects look like and why they warrant ethical attention. Empirical studies of the kinds cited above will be part of filling in this picture, but they cannot achieve it without a clear conceptual and normative framework, which characterises the nature of our interests in developing and inhabiting our identities and the roles of information in this, through which to interpret their findings.

Here I have addressed these gaps by proposing a normative account of the role of information about our brains and minds in narrative self-constitution. I have argued that these kinds of information can help us to construct identity-constituting narratives that make sense of our experiences and support us in navigating our lives. It does so not only by providing characteristics and plotlines for these narratives, but also by offering valuable explanatory and interpretive context for our embodied experiences – experiences of ill health, shifting moods, and changing cognitive and physical capacities. Not all information generated by neurotechnologies will fulfil these roles equally well, or in the same ways for everyone. However, to the extent that that it does, it offers a vital tool in the construction of coherent and inhabitable identity-constituting narratives.

The context-dependent and often idiosyncratic ways in which information about our brains and minds generated by neurotechnologies impacts upon our self-narratives means that governing this information so as to protect our identity-related interests may not be a straightforward matter. But this alone is not reason to ignore them. I have argued that, because our identity-constituting narratives provide the foundations for our self-understanding, values and practical engagement with the world, we have critical interests developing coherent and inhabitable self-narratives, and thus in the epistemic tools for doing so. This includes information about our brains and minds. For these reasons, I have argued, that decisions and practices governing whether and, crucially, how we have access to information generated by neurotechnologies must take into account the extent to which this supports or undermines these interests. And where information generated by neurotechnologies engages these interests, they warrant comparable attention alongside more commonly considered information-related concerns such as impacts on health and autonomy, and risks to privacy.

## Data Availability

Not applicable.

## Footnotes

1 There is considerable body of scholarship discussing the effects of the presence or operation of implanted BCIs, neuroprosthetics and neuromodulation devices (especially the cognitive, affective and behavioural effects of deep brain stimulation (DBS)) on users’ sense of embodiment or identity (see, for example, [1–5]). In some circumstances it may not be possible to draw a neat line between the effects of information and the effects of the presence or operation of a device. Though the focus here is on the former, there is likely to be value in further ethical exploration of the relative contributions and interaction between these different impacts.

2 References to ‘users’ here is intended to refer to those who use neurotechnologies for their own purposes or are subject to uses of neurotechnologies by others (for example radiographers administering brain imaging), not the parties who administer them. I shall also use the phrase ‘information subjects’ to refer to this group.

3 Which regulatory regime or governance approach applies may also be ambiguous. For example, technologies such as mental wellbeing apps are ambiguously situated between the domains of healthcare and consumer entertainment.

4 In parallel to the position I set out here, I have argued elsewhere that genetic may play a significant role in our identity-constituting narratives, and also that the ways that identity, and the normative aspects of the relationship between identity and genetic information, are not always well-conceived or fully articulated in existing discussions – for example, where these appeal to, or are assumed to appeal to, a geneticised view of the identity as pre-determined by genetic traits or parentage [33, 34]

5 Though it is not the focus of the present discussion, neuroethical debates are appropriately concerned with identity in this numerical sense, for example in circumstances where neurodata is sufficiently rich that its analysis reveals its source as a particular individual (‘identifies’ one with the other) thus, permitting the attribution of traits or disorders revealed by the data to this individual, undermining their privacy and placing them at risk of stigma or discrimination. This kind of identification may be a particular risk where machine learning is used to analyse large quantities of data [23].

6 In these respects, the account proposed here will differ from those that those that argue that almost any change to one’s self-narrative is a morally neutral part of narrative development, or that any concerns about narrative impacts are at root those about harm to autonomy (see, for example, [39, 40]).

7 Proponents of narrative theories of identity differ as to the extent to which our narratives should be seen as first-personal, co-authored by others, or strike a balance between our own and others’ perspectives on who we are (for example, Françoise Baylis offers an account of this last kind [45]).

8 Narrative ‘coherence’ here refers to an ideal, rather than a readily achievable state. Minimising internal contradictions and tensions is valuable insofar as our identities provide a consistent perspective from which to interpret, judge, act. However, the complexities of most lives make eradication of all such features unlikely if not impossible and any coherence achieved is likely to be imperfect as well as, in Mackenzie’s and Walker’s words, “dynamic and provisional” [40 p.381].

9 It seems likely that there will be a degree of interdependence as well as mutual delimiting between these four considerations. For example, achieving coherence between one’s narrative and our lived experiences will help to sustain the narrative’s internal coherence insofar as it includes aspects of these experiences.

10 Schechtman’s account in ‘The Constitution of Selves’ relegates the relevance of our bodies to questions of numerical identities insofar as they allow other people to reidentify us [42].

11 Schechtman is not alone in presenting a peculiarly rationalist – relational, but disembodied – view of narrative self-constitution, similar observations may be made of the accounts proposed by Charles Taylor and Alasdair Macintyre [46, 54].

12 With the exception perhaps of serious cognitive and psychiatric conditions, the ways and extent to which embodied states and experiences impact on our capacities to construct coherent, inhabitable self-narratives is not inherent, but rather it seems likely to depend to a large degree on what particular embodied characteristics – such as our gender or (dis)ability – means in any particular social and cultural context and this what this implies for the narratives available to us and the recognition of those narratives by others (for further discussion, see [47]).

13 Although I have described it as ‘straightforward’ here, the very nature of narratives – as interpretive wholes that change and are changed by the meaning of their parts – means that it is unlikely that a change of self-descriptor would ever be just that, a discrete edit without wider interpretive ramifications.

14 Objections to bio-essentialist views of identity – as both empirically unsound and morally objectionable – are familiar from ethical debates about geneticisation and the identity-significance of genetic information [31, 58]. These debates, however, sometimes make unwarranted assumptions that any claim to identity-value must be grounded the in claims about the essential value of genetic information, rather than the kind of contingent, instrumental value proposed here.

15 Of all categories of biological or health-related information, the identity relevance of genetic information has perhaps received the greatest attention in bioethical, philosophical, social science and legal literature, and in the law, particularly where entitlements to knowledge of genetic illness and genetic parentage are concerned, and in the context of direct-to-consumer genetic testing (see, for example, 30–32). However, while the *qualitative* impacts of genetic information on identity are widely discussed, the normative significance of its role the construction of practical identity narratives remains largely unexplored, with the notable exception of some analyses of knowledge of genetic parentage [see, for example, 59, 60].

16 I have argued elsewhere that a wide variety of personal bioinformation about different aspects of our bodily and biological attributes and relationships, generated in many different contexts could play ethically significant – though contingent – roles in the construction of our self-narratives, and provided examples of the variety of ways in which they might do so (see, [33, 34]).

17 Lisa Bortolotti has made similar claims in suggesting that reliable information about our own minds, such as that generated through psychological experiments, is “necessary for self-authorship” because it allows us to develop more “accurate” self-narratives that align with more closely with our actual attitudes and motivations [64, p.687].

18 The kind of ‘truth’ claims rejected here are those relating to truth about one’s identity. It is of course possible that someone could wish to learn the truth about an aspect of her body or health – for example the state of brain damage she sustained following trauma – in order to construct her self-narrative. Seeking this kind of truth is not only compatible with, but central to, the claims made here.

19 A number of authors are sceptical about the possibility of or threat posed by mind-reading, citing not only the potentially contingent capabilities of current technologies, but also the category mistake of equating knowledge of neural activity with knowledge of the content and qualities of our thoughts (see, for example, [6, 69, 70]).

20 Distinctions between the preservation of identity and how welcome or valuable such preservation is are discussed and illustrated in the literature discussing the behavioural and personality impacts of deep brain stimulation (see, for example, [39, 72, 73].

21 One example of this, albeit from a different context, may be found in some studies investigating the reactions of some donor-conceived adults to learning of their donor parentage where despite the initial distressing disruption of their existing self-conception they value having this knowledge [74]).

22 The information provided by such devices is a visual/sound alert rather than detailed verbal output. However, its semantic content can be understood as extending to how this alert is interpreted by the user in conjunction with, for example, guidance from their clinicians about what it indicates and how they should respond, as well as their own understanding of managing their epilepsy. This is reflected in the language used, for example, by Gilbert and co-authors when they refer to these alerts as “predictive and advisory” [78 p. 4].

23 Although the authors use the term “self-narrative” here, this does not signal that they employ a theoretical framework of narrative self-constitution. Rather, in accordance with to the phenomenological qualitative research methods used in the study, this term appears to be used simply to refer to the account that participants give of their own experiences [38].

24 Françoise Baylis makes a similar point with respect to effects on identity of personality changes accompanying treatment using deep-brain stimulation, though the extent and nature of change that Baylis regards as ethically innocuous is wider than that proposed here [39].

25 DBS is another context in which the authors have discussed the ethical ramifications of self-estrangement, in that context, precipitated by behavioural and personality changes that occur following direct electronic stimulation of neurones by implanted electrodes [see, for example 80].

26 This example further highlights (see n1 above) how it may not always be possible to separate the users’ experiences of, and the identity impacts, of their interaction with the device itself and their encounters with the information generated by the device. This also underscores what I say below about the importance of attending to the manner in information is communicated.

27 For example, because of the risks of rendering someone’s self-narrative unrecognisable, incoherent or uninhabitable, consideration of identity interests is likely to recommend a more limited range of information disclosure than approaches that equate autonomy interests with either ‘whatever the individual wants to know’ or with the idea that as long as information is reliable it can only benefit our capacities for autonomy by equipping us with additional material on which to reflect and base our decisions [90, 91]. Meanwhile, considerations of identity may recommend wider grounds for disclosing neuro-information than those which equate autonomy interests with informational control or protection against unsought information, as it recognises the potential identity value of as-yet unknown information [92].

28 The phrase ‘individually relevant’ is used to distinguish findings that are about the circumstances of a particular participant from aggregated research results.

29 This kind of argument has been advanced most notably in relation to access to direct-to-consumer genomic testing where the clinical validity and utility of some tests are questionable [91].

30 For example, studies conducted with research participants undergoing genetic testing for variants associated with increased risk of developing late onset Alzhemer’s disease indicate that genetic counselling about the limited predictive power of the findings mitigated (though did not wholly obviate) some of the ways that participants viewed the findings and used them to reflect upon their lives, relationship and priorities [95].

31 It seems likely that information recipients’ appreciation of the epistemic limitations of the information is not only shaped at the ‘point of use’ or disclosure, but, as noted above, also by wider cultural discussions about the nature and value of information generated by neurotechnologies.

32 The authors clarify that exclusion from early experimental trials on grounds of identity risks would not deny participants access to proven therapeutic interventions.
